# A New Combination of *Bifidobacterium bifidum* and *Lactococcus lactis* Strains with Synergistic Effects Alleviates Colitis-Associated Colorectal Cancer

**DOI:** 10.3390/foods13193054

**Published:** 2024-09-25

**Authors:** Jiacui Shang, Lijun Liu, Shuo Yang, Bofan Duan, Shuiqi Xie, Xiangchen Meng

**Affiliations:** 1 Key Laboratory of Dairy Science, Ministry of Education, Northeast Agricultural University, Harbin 150030, China; sjc1224@126.com (J.S.); 17806286824@163.com (L.L.); yangshuo8023@163.com (S.Y.); dbf02211204@163.com (B.D.); xsq17889982102@163.com (S.X.); 2 Food College, Northeast Agricultural University, Harbin 150030, China

**Keywords:** probiotics, chronic inflammation, tight junction protein, gut microbiota

## Abstract

Chronic inflammation is a factor in the development of cancer, and probiotics play a role in preventing or treating inflammation as an adjuvant therapy. To investigate potential probiotics for the prevention of colitis-associated colorectal cancer (CAC), *Bifidobacterium bifidum* H3-R2 and *Lactococcus lactis* KLDS4.0325 were used to examine the effects on colon cancer cells and in an inflammation-related cancer animal model. The results revealed that *B. bifidum* H3-R2 in combination with *L. lactis* KLDS4.0325 caused apoptosis in colon cancer cells by increasing caspase-3 and caspase-9 protein levels, enhancing Bax expression, and lowering Bcl-2 expression. In addition, the combination of the two strains relieved the tissue damage; reduced proinflammatory cytokines, myeloperoxidase (MPO) activity, and hypoxia-inducible factor 1-alpha (HIF-1α) level; upregulated anti-inflammatory cytokines; increased colonic tight junction protein expression; regulated intestinal homeostasis by inhibiting NLRP3 inflammasome signaling pathway; and improved the imbalance of gut microbiota in animal models. Moreover, the combination of the two strains had a greater preventive impact than each strain alone. These findings are supportive of clinical studies and product development of multi-strain probiotic preparations for diseases associated with colitis.

## 1. Introduction

The majority of bowel cancers are closely associated with inflammatory bowel disease (IBD), which includes Crohn’s disease and ulcerative colitis (UC). Chronic inflammation may result in an increase in tissue proliferation, which may then result in the development of tumors [1]. Colitis-associated colorectal cancer is a typical type of cancer caused by inflammation, and the relationship between UC and colitis associated-cancer (CAC) has been shown [2]. According to studies, patients with UC have a substantially greater incidence of colorectal cancer (CRC) than the general population, up to 18%, 30 years after the disease first manifests [3]. The association between inflammation and tumors has recently become a focus of research to better understand the occurrence and progression of tumors. Therefore, it is essential to control disease activity, maintain long-term remission, and prevent carcinogenesis.

The public’s understanding of the function of probiotics in the prevention, alleviation, and therapy of specific diseases has been improved due to recent research on intestinal microbiota [4]. Probiotics can change the composition of gut microbiota [5], compete with pathogenic microorganisms, improve the host immune response, inhibit the inflammatory response, and repair the function of the injured mucosal barrier to prevent intestinal inflammation [6]. Some probiotics have been utilized in clinical practice, mostly for postoperative intestinal protection. Additionally, probiotics are used to reduce the body’s resistance to conventional CRC drugs [7]. Because of the different properties of strains, single-strain and multi-strain probiotics have distinct impacts on inflammation and associated diseases. Currently, the remission effects of *Bifidobacterium* and *Lactobacillus* on CAC are mostly explored, but the effect of *L. lactis* on CAC is rarely studied.

Our laboratory previously isolated a strain of *L. lactis* KLDS4.0325 from Xinjiang traditional dairy products, and the fermented milk containing *L. lactis* KLDS4.0325 had the effect of regulating the intestinal flora of mice [8]. Additionally, earlier studies have demonstrated that *B. bifidum* H3-R2 can repair damaged intestinal mucosa and reduce inflammation [9]. To develop probiotics with CRC-preventive effects, the proliferation-inhibiting and pro-apoptotic influences of *B. bifidum* H3-R2 and *L. lactis* KLDS4.0325 on colon cancer HT-29 cells were evaluated. Additionally, the impacts of the strains on inflammatory cytokines, intestinal barrier function, and intestinal flora composition in CRC-affected mice were analyzed.

## 2. Materials and Methods

### 2.1. Cells and Bacterial Strains

*B. bifidum* H3-R2 was anaerobically incubated in a De Man, Rogosa, and Sharpe (MRS; Oxoid, Basingstoke, United Kingdom) medium containing 0.05% L-cysteine hydrochloride (mMRS), and *L. lactis* KLDS4.0325 was aerobically incubated in GM17 broth (Qingdao Haibo Biotechnology Co., Ltd., Qingdao, China) at 37 °C for 16 h. After obtaining bacterial cells by centrifugation (8000× *g* for 5 min at 4 °C), the experiment’s concentration was adjusted. In the mixed bacterial suspension, *B. bifidum* H3-R2 and *L. lactis* KLDS4.0325 were mixed in a ratio of 1:1. Human colon cancer HT-29 cells were acquired from the Shanghai Institute of Cell Biology (Shanghai, China) and cultured with fetal bovine serum (10%) in Dulbecco’s modified Eagle’s medium (DMEM, Gibco Life Technologies, Grand Island, NY, USA). Cultures were performed at 37 °C in a 5% CO_2_ and 95% filtered air atmosphere.

### 2.2. Hoechst Staining Assay

The HT-29 cells were cultured for 24 h in 6-well plates with 1 × 10^5^ per well. The cells were incubated with *L. lactis* KLDS4.0325 and/or *B. bifidum* H3-R2 (MOI =10) for 12 h. As a positive control group, five-Fu (Shanghai Aladdin Bio-Chem Technology Co., Ltd., Shanghai, China) was treated with HT-29 cells. Cultures were performed at 37 °C in a 5% CO_2_ and 95% filtered air atmosphere. As a blank control group, HT-29 cells that had not been exposed to bacteria were used. Following 12 h of incubation, the culture medium was withdrawn, and a fixative of 0.5 mL was applied. It was then incubated for 10 min and rinsed with PBS twice. Following that, the staining solution Hoechst 33258 was applied, and the cells were allowed to stain for 5 min. Following the removal of the staining solution, PBS was applied twice. After the addition of the antifluorescence quenching liquid, observation was carried out using a fluorescence microscope [10].

### 2.3. Cell Viability Assay

The proliferation and viability of HT-29 cells with *L. lactis* KLDS4.0325 and/or *B. bifidum* H3-R2 were evaluated using CCK-8. The HT-29 cell treatment protocol was identical to that employed in the morphology analysis of apoptosis in these cells. The culture medium was taken out of the wells and washed with PBS three times after 6 and 12 h of incubation. CCK-8 (10 μL) and DMEM (100 μL) were then added, and the mixture was incubated for 2 h. At 450 nm, the absorbance was determined [11].

### 2.4. Evaluation of HT-29 Cell Apoptosis by Flow Cytometry

The HT-29 cell treatment protocol was identical to that employed in the morphology analysis of apoptosis in these cells. After transferring the mixture from the 6-well plates to a centrifuge tube with a capacity of 15 mL, it was then washed with 1 mL of PBS. After 5 min of centrifugation at 1000× *g* and removal of the supernatant, the residual cells were gently resuspended in a binding solution containing annexin V-fluorescein isothiocyanate (FITC, 195 µL), after which the remaining annexin V-FITC (5 µL) was added. The mixture was then carefully mixed with propidium iodide stain (10 µL). The cells were subjected to incubation in the dark at room temperature for 20 min, placed in centrifuge tubes, and detected by flow cytometry.

### 2.5. Quantitative Real-Time PCR (qRT-PCR)

According to the manufacturer’s recommendations, total RNA from the colon’s tissues and cells was extracted using an RNA extraction kit, and 1 μg of the extracted RNA was then reverse-transcribed into cDNA utilizing a FastKing gDNA Dispelling RT SuperMix kit. The SYBR Green Master Mix and Step One Plus Real-Time PCR system (Applied Biosystems, Carlsbad, CA, USA) were employed to conduct the qRT-PCR evaluation. Relative expressions of genes were determined utilizing the comparative threshold cycle (2^−ΔΔCt^) method [12,13]. GAPDH and β-actin were utilized to normalize gene expression in cells or animal tissue, respectively.

### 2.6. Animals and Experimental Design

Sixty male C57 BL/6 J mice (5 weeks old) were acquired from the Beijing Vital River Laboratory Animal Technology Co., Ltd. (Beijing, China). Mice were kept for one week in a laboratory setting with regulated conditions, including a temperature of 23 ± 2 °C, a lighting schedule consisting of light–dark cycles lasting 12 h each, and unrestricted access to food and water. The mice were then separated into six groups (*n* = 10/group): the normal control group (NC), model group (MC), positive control group (PC), *B. bifidum* H3-R2 group (BH), *L. lactis* KLDS4.0325 group (LK), and both *B. bifidum* H3-R2 and *L. lactis* KLDS4.0325 group (BL). Mice were given intraperitoneal injections of 10 mg/kg azoxymethane (AOM; Sigma, Rehovot, Israel) and 2.5% DSS (molecular weight of 36–50 kDa, MP Biomedical, Solon, OH, USA) in the drinking water twice (days 8–12 and 23–27) for the AOM/DSS study [14]. Throughout the experiment, the BH, LK, and BL groups received 200 μL of *B. bifidum* H3-R2 suspension (1 × 10^9^ CFU/mL), *L. lactis* KLDS4.0325 suspension (1 × 10^9^ CFU/mL), and a mixed suspension of *B. bifidum* H3-R2 and *L. lactis* KLDS4.0325 (1 × 10^9^ CFU/mL) via oral gavage once daily. PC group was orally administered 200 μL of 5-ASA every day. Equal volumes of physiological saline were given once daily orally to the MC and NC groups. The weight of mice was recorded at the same time every 5 days. At the end of the tests, anesthesia was utilized to euthanize the mice. The Northeast Agricultural University’s Institutional Animal Care and Use Committee approved all animal experiments.

### 2.7. Organ Index

Mice were weighed prior to their sacrifice. Rapid aseptic removal of the spleen was performed. The formula below was utilized to compute the spleen index: organ index (mg/g) = organ weight/body weight.

### 2.8. Histopathologic Analysis

Colon tissue was fixed with paraformaldehyde (4%) and then rinsed and dehydrated. The samples were embedded with paraffin, cut into 4 μm sections, dewaxed, and stained with hematoxylin-eosin (H&E). Finally, the histopathological changes to various groups of pancreatic tissue were examined under a 200× microscope (Olympus BX53, Olympus, Tokyo, Japan) [15].

### 2.9. Evaluation of Myeloperoxidase (MPO) Activity and Hypoxia-Inducible Factor 1-Alpha (HIF-1α) Level

Ground colon tissue samples were homogenized at a ratio of 1:19 (*w*/*v*) with reagents from an MPO assay kit (Chenglin Bioengineering Institute, Beijing, China), and MPO activity was then assessed in accordance with the guidelines. The HIF-1α level in colon tissues was detected using the HIF-1α kit (Chenglin Bioengineering Institute, Beijing, China).

### 2.10. Cytokine Levels Analysis

Colon tissue samples were homogenized in 1:9 (*w*/*v*) sterile PBS and centrifuged (7000× *g* at 4 °C for 15 min). Interleukin-10 (IL-10), IL-6, IL-17, IL-1β, IL-22, and tumor necrosis factor-α (TNF-α) levels in the supernatant were measured using ELISA kits (Chenglin, Beijing, China) according to the manufacturer’s guidelines.

### 2.11. 16 S rRNA Sequencing

The QIAamp PowerFecal DNA Kit (QIAGEN) was employed to extract the fecal genomic DNA. In a PCR system, the amplification of the bacteria’s 16 S rRNA gene’s V3-V4 hypervariable regions was carried out utilizing the primers 806 R (5′-GGACTACHVGGGTWTCTAAT-3′) and 338 F (5′-ACTCCTACGGGAGGCAGCAG-3′). Majorbio Bio-Pharm Technology Co. Ltd. (Shanghai, China) performed paired-end sequencing on an Illumina MiSeq platform. After removing chimeric reads, the remaining cleaned sequences that met the UPARSE pipeline’s similarity threshold of at least 97% were clustered into operational taxonomic units (OTUs). Based on the observed OTU abundance, a principal component analysis (PCA) was carried out utilizing the R vegan package. Heat maps of bacterial species were created using the R pheatmap package. The QIIME 2 2024.5 software was utilized to classify OTU species through comparison with the database. Additionally, a histogram of species was created for each sample within the classification levels to analyze the various species’ proportions [16].

## 3. Results

### 3.1. Examination of Apoptosis Morphology in HT-29 Cell

**Figure 1:**
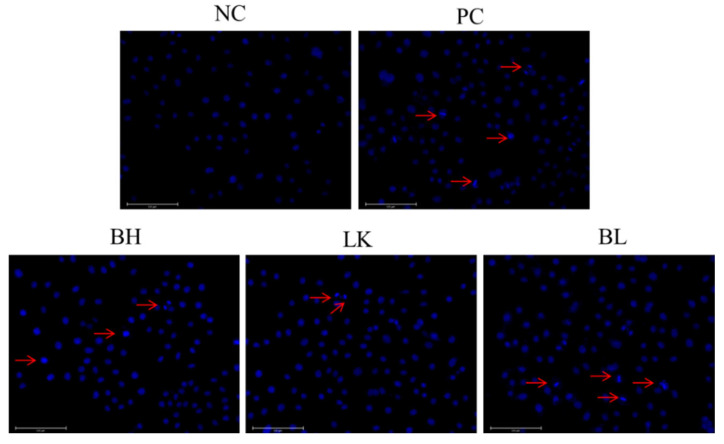
Morphological features of the HT-29 cells treated with *B. bifidum* H3-R2 and *L. lactis* KLDS4.0325 after 12 h. NC, normal control group; PC, 5-FU group; BH, *B. bifidum* H3-R2 group; LK, *L. lactis* KLDS4.0325 group; BL, combination of *B. bifidum* H3-R2 and *L. lactis* KLDS4.0325 group. A fluorescence microscope was used to photograph images (20×). The red arrows indicate the corresponding apoptosis cells.

Cell morphology was observed by Hoechst 33258 staining (Figure 1). Normal cells’ nuclei were stained as ordinary blue, whereas apoptotic cells’ nuclei were bright blue. The combined strain group, which was comparable to the positive control group, displayed a brighter blue fluorescence in the nuclei of HT-29 cells than the normal group. In HT-29 cells, the combination of *B. bifidum* H3-R2 and *L. lactis* KLDS4.0325 induced apoptosis.

### 3.2. Cell Viability

**Figure 2:**
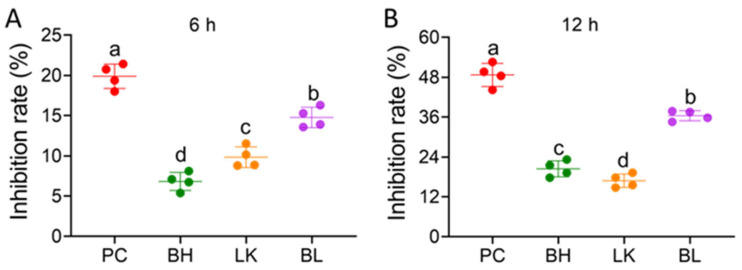
Effect of *B. bifidum* H3-R2 and *L. lactis* KLDS4.0325 on proliferation level of HT-29 cells for 6 h (**A**) and 12 h (**B**). PC, 5-FU group; BH, *B. bifidum* H3-R2 group; LK, *L. lactis* KLDS4.0325 group; BL, combination of *B. bifidum* H3-R2 and *L. lactis* KLDS4.0325 group. All data are expressed as mean ± SD. Different letters represent significant differences between different groups (*p* < 0.05).

The bacterial strains inhibited the growth of HT-29 cells, and the effect became stronger as the incubation period was prolonged (Figure 2). After 6 h and 12 h of incubation with HT-29 cells, the inhibition rate of *B. bifidum* H3-R2 combination with *L. lactis* KLDS4.0325 was 14.78% and 36.42%, respectively. Combination strain treatments resulted in much higher inhibition rates than single-strain treatments but significantly lower inhibition rates than the positive control group (*p* < 0.05).

### 3.3. Assaying of Apoptosis

**Figure 3:**
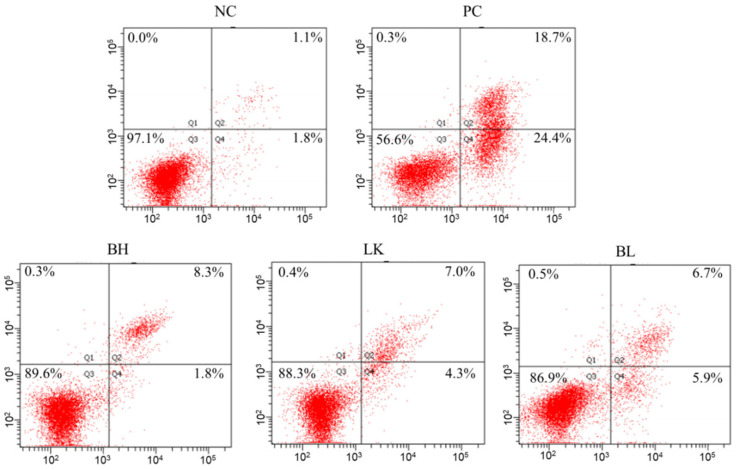
Effect of *B. bifidum* H3-R2 and *L. lactis* KLDS4.0325 on apoptosis rate of HT-29 cells. NC, normal control group; PC, 5-FU group; BH, *B. bifidum* H3-R2 group; LK, *L. lactis* KLDS4.0325 group; BL, combination of *B. bifidum* H3-R2 and *L. lactis* KLDS4.0325 group.

Flow cytometry was employed to assess the apoptosis rate of HT-29 cells after a 12 h incubation period (Figure 3). With the exception of the normal group, all groups had increased numbers of apoptotic cells in the Q2 and Q4 portions. When HT-29 cells were co-cultured with *B. bifidum* H3-R2 and *L. lactis* KLDS4.0325, the apoptotic rates were 10.1% and 11.3%, respectively. The rate of apoptosis in the combined strain group was 12.6%, which was lower than the positive control group (43.1%; *p* < 0.05).

### 3.4. Expression of Genes and Proteins Related to Apoptosis in HT-29 Cells

**Figure 4:**
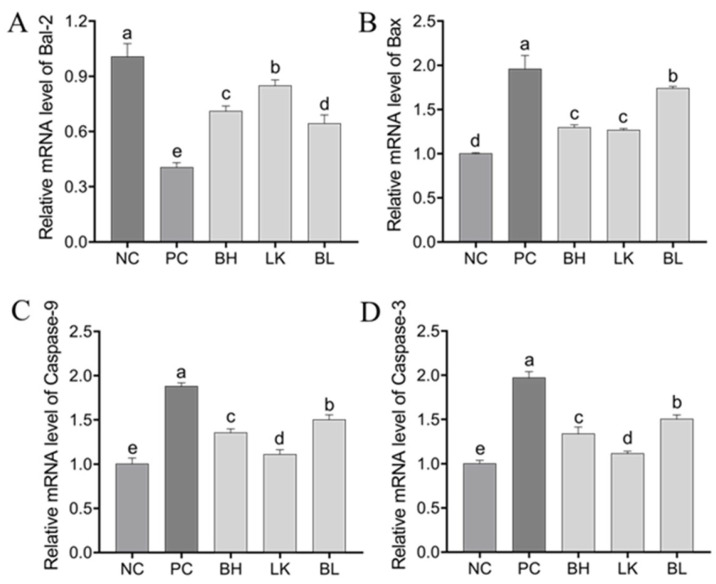
Effect of *B. bifidum* H3-R2 and *L. lactis* KLDS4.0325 on apoptosis associated proteins in HT-29 cells. (**A**) Relative Bal-2 mRNA expression; (**B**) Relative Bax mRNA expression; (**C**) Relative caspase-9 mRNA expression; (**D**) Relative caspase-3 mRNA expression. NC, normal control group; PC, 5-FU group; BH, *B. bifidum* H3-R2 group; LK, *L. lactis* KLDS4.0325 group; BL, combination of *B. bifidum* H3-R2 and *L. lactis* KLDS4.0325 group. All data are expressed as mean ± SD. Different letters represent significant differences between different groups (*p* < 0.05).

In comparison to the NC group, all other groups’ relative expressions of caspase-3, caspase-9, and Bax mRNA were dramatically elevated (*p* < 0.05), whereas BAL-2 expression was significantly downregulated (*p* < 0.05; Figure 4). In all strain treatment groups, the combined strain group had the most substantial impact on controlling protein expression (*p* < 0.05).

### 3.5. Pathogenesis

**Figure 5:**
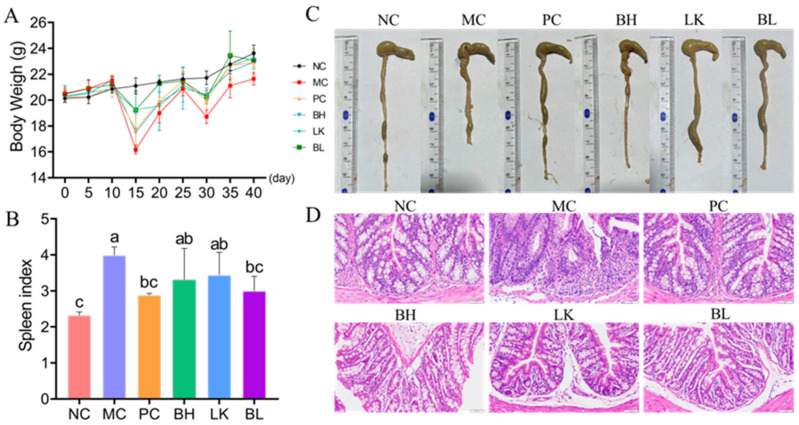
Effects of *B. bifidum* H3-R2 and *L. lactis* KLDS4.0325 administration on body weight (**A**), spleen indexes of thymus (**B**), colon length (**C**), and histopathological observation of the colon (magnification ×200) (**D**) in mice. NC, normal control group; MC, AOM/DSS-induced model group; PC, 5-FU group; BH, *B. bifidum* H3-R2 group; LK, *L. lactis* KLDS4.0325 group; BL, combination of *B. bifidum* H3-R2 and *L. lactis* KLDS4.0325 group. All data are expressed as mean ± SD. Different letters represent significant differences between different groups (*p* < 0.05).

The mice in the MC group had significantly less body weight than the mice in the NC group at the end of the experiment (*p* < 0.05; Figure 5A). After giving AOM/DSS mice a combination of *B. bifidum* H3-R2 and *L. lactis* KLDS4.0325, the mice’s body weight was recovered (*p* < 0.05), and there was no discernible difference between them and the positive control group (*p* > 0.05). When compared to the NC group, the MC group’s spleen index was substantially higher (Figure 5B). The mice’s spleen index was considerably lowered in the BL group when compared to the MC group (*p* < 0.05). The spleen index of the NC, PC, and BL groups did not differ significantly (*p* > 0.05).

In comparison to the normal group, the MC group’s colon length was considerably shorter (*p* < 0.05, Figure 5C). The length of the colons in the BH, LK, and BL groups was shorter than in the NC and PC groups but significantly longer than in the MC group (*p* < 0.05). AOM/DSS treatment led to the destruction of the colonic epithelial structure, the infiltration of inflammatory cells, the reduction of goblet cells, the shortening of the crypt, and the formation of loose arrangements (Figure 5D). Compared to the MC group, the strain treatment groups had reduced levels of inflammatory cell infiltration, and the crypt morphology was relatively uniform.

### 3.6. MPO Activity and HIF-1α Levels

**Figure 6:**
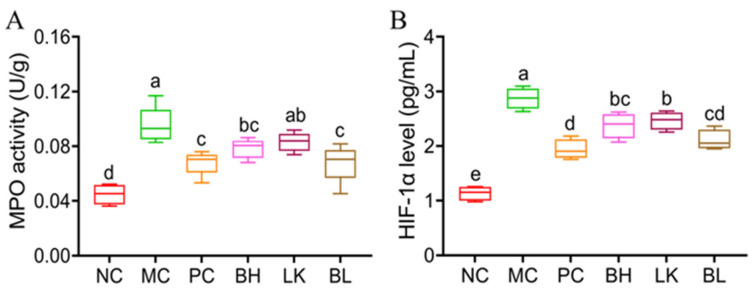
Effects of *B. bifidum* H3-R2 and *L. lactis* KLDS4.0325 administration on MPO activity (**A**), and HIF-1α level (**B**) of colons in mice. NC, normal control group; MC, AOM/DSS-induced model group; PC, 5-FU group; BH, *B. bifidum* H3-R2 group; LK, *L. lactis* KLDS4.0325 group; BL, combination of *B. bifidum* H3-R2 and *L. lactis* KLDS4.0325 group. All data are expressed as mean ± SD. Different letters represent significant differences between different groups (*p* < 0.05).

As demonstrated in Figure 6A, the MPO activity of colon tissue in the MC group rose considerably following AOM/DSS treatment (*p* < 0.05). The BH and BL groups significantly reduced the colonic MPO activity of MC mice (*p* < 0.05), but there was no significant difference with the PC group. AOM/DSS treatment significantly increased (*p* < 0.05) the level of HIF-1α (Figure 6B). Compared with the MC group, HIF-1α levels in BH, LK, and BL groups were significantly decreased (*p* < 0.05).

### 3.7. Cytokine Levels

**Figure 7:**
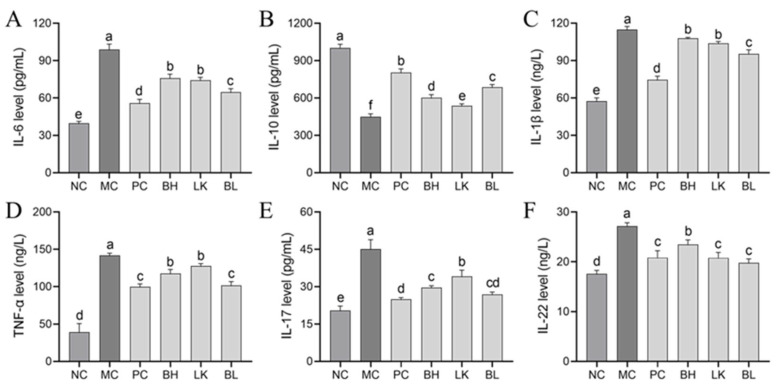
Effects of *B. bifidum* H3-R2 and *L. lactis* KLDS4.0325 administration on the secretion of IL-6 (**A**), IL-10 (**B**), IL-1β (**C**), TNF-α (**D**), IL-17 (**E**), and IL-22 (**F**) in mice. NC, normal control group; MC, AOM/DSS-induced model group; PC, 5-FU group; BH, *B. bifidum* H3-R2 group; LK, *L. lactis* KLDS4.0325 group; BL, combination of *B. bifidum* H3-R2 and *L. lactis* KLDS4.0325 group. All data are expressed as mean ± SD. Different letters represent significant differences between different groups (*p* < 0.05).

In the MC group, IL-1β, IL-6, IL-22, IL-17, and TNF-α concentrations were considerably higher (*p* < 0.05) than in the NC group, whereas IL-10 concentration was significantly lower (Figure 7). Lower IL-1β, IL-6, IL-17, TNF-α, and IL-22 concentrations were observed in the strain treatment groups compared to the MC group, and no significant differences were observed in the TNF-α, IL-17, and IL-22 levels between the PC and BL groups (*p* > 0.05).

### 3.8. Tight Junction Protein Expression

**Figure 8:**
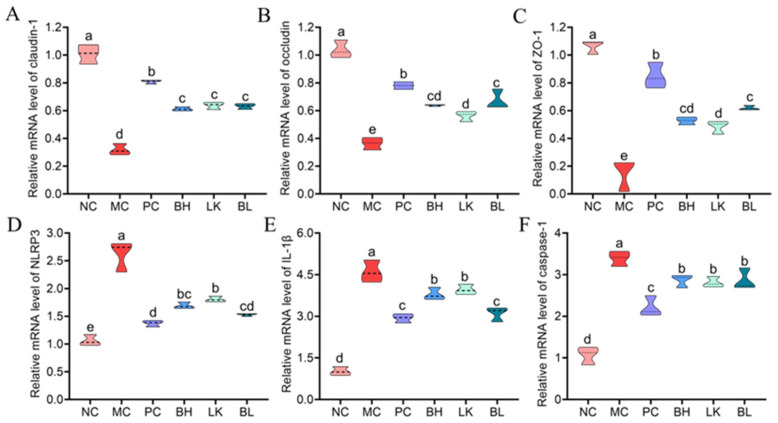
Effects of *B. bifidum* H3-R2 and *L. lactis* KLDS4.0325 administration on the mRNA levels of claudin-1 (**A**), occludin (**B**), ZO-1 (**C**), NLRP3 (**D**), IL-1β (**E**), and caspase-1 (**F**) in colon. NC, normal control group; MC, AOM/DSS-induced model group; PC, 5-FU group; BH, *B. bifidum* H3-R2 group; LK, *L. lactis* KLDS4.0325 group; BL, combination of *B. bifidum* H3-R2 and *L. lactis* KLDS4.0325 group. All data are expressed as mean ± SD. Different letters represent significant differences between different groups (*p* < 0.05).

Occludin, claudin-1, and ZO-1 relative mRNA expressions were all considerably lower (*p* < 0.05) in the MC group than in the NC group (Figure 8A–C). The relative mRNA expressions of ZO-1, occludin, and claudin-1 in the BH, LK, and BL groups were considerably greater (*p* < 0.05) than in the MC group. The occludin and ZO-1 levels did not differ significantly between the BH and BL groups, but they were considerably higher than in the LK group.

### 3.9. NLRP3 Inflammasome Activation

In the MC group, AOM/DSS activated IL-1β, NLRP3, and caspase-1 expression (Figure 8D–F). In comparison to the MC group, the BH, LK, and BL groups had lower levels of NLRP3, IL-1β, and caspase-1 (*p* < 0.05), while there was no discernible difference in caspase-1 levels between the strain treatment groups. In comparison to the PC group, the NLRP3 and IL-1β expressions were similar in the BL group (*p* > 0.05).

### 3.10. Composition of Gut Microbiota

**Figure 9:**
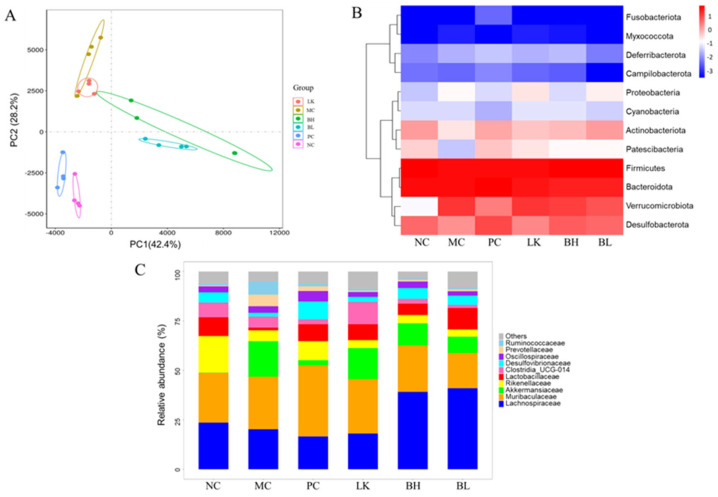
Effects of *B. bifidum* H3-R2 and *L. lactis* KLDS4.0325 administration on the gut microbiota. (**A**) PCA analysis of bacterial community structure; (**B**) correlation heatmap at the phylum level; (**C**) relative abundance of microbiota at the family level. NC, normal control group; MC, AOM/DSS-induced model group; PC, 5-FU group; BH, *B. bifidum* H3-R2 group; LK, *L. lactis* KLDS4.0325 group; BL, combination of *B. bifidum* H3-R2 and *L. lactis* KLDS4.0325 group. All data are expressed as mean ± SD. Different letters represent significant differences between different groups (*p* < 0.05).

Following clustering at a 97% similarity threshold, 11317 operational taxonomic units were identified for analysis. The differences in microbiome composition between those groups were assessed using principal component analysis, with the first and second principal components accounting for 42.4% and 28.2% of the observed diversity, respectively (Figure 9A).

At the phylum level, the predominant gut microbiota phyla in the NC group were Firmicutes, Bacteroidetes, Desulfobacterota, and Actinobacteriota (Figure 9B). In the MC group, AOM/DSS treatment substantially enhanced Verrucomicrobiota and decreased Desulphurobacteria and Actinobacteria. BH and BL intervention could reduce the abundance of Verrucomicrobiota induced by AOM/DSS. At the family level, Muribaculaceae, Lachnospiraceae, Rikenellaceae, and Lactobacillaceae are the predominant gut microbiota phyla for the NC group (Figure 9C). In the MC group, AOM/DSS treatment decreased *Lactobacillaceae* and *Rikenellaceae* and increased *Akkermansiaceae*. In comparison to the MC group, the relative abundance of Lactobacillaceae was significantly higher in the BH, LK, and BL groups, particularly in the BL group.

## 4. Discussion

CRC is a disease that has a high morbidity as well as a high mortality rate. There are currently a number of drugs available for the treatment of CRC. The role of probiotics in the prevention and adjuvant treatment of CRC has attracted a lot of attention recently, and probiotic supplements have been proposed as a potential new treatment strategy [17,18,19]. Thirabunyanon et al. found that *Enterococcus faecium* and *Lactobacillus fermentum* derived from fermented milk could inhibit the proliferation of Caco-2 cells [20]. Shi et al. discovered that *Lactobacillus paracasei* extracellular vesicles could trigger apoptosis and inhibit the growth of CRC cells by activating the PDK1/AKT/Bcl-2 signaling pathway [21].

Apoptosis is the autonomic and orderly death of cells controlled by genes. Controlling caspase and Bcl-2 protein expression can inhibit the proliferation of cancer cells and induce apoptosis [22,23]. Bax, a pro-apoptotic effector protein, is responsible for increasing the permeability of the mitochondrial membrane and releasing cytochrome C into the cytoplasm, whereas Bcl-2, an anti-apoptotic effector protein, can inhibit this process [24]. After being released from the mitochondria and entering the cytoplasm, cytochrome C binds to the apoptotic protease activator to activate caspase-9 and caspase-3, which ultimately causes apoptosis. In HT-29 cells, treatment with *B. bifidum* H3-R2 and *L. lactis* KLDS4.0325 resulted in a decrease in Bcl-2 expression and an increase in Bax, caspase-3, and caspase-9 expression, suggesting that H3-R2 and KLDS 4.0325 may be able to control the levels of caspase and Bcl-2 family proteins to promote colon cancer cell apoptosis through the mitochondrial pathway.

Both *B. bifidum* H3-R2 and *L. lactis* KLDS4.0325 were capable of inducing apoptosis in colon cancer cells in vitro. The strains’ effect on CAC in animal models was further investigated. Intestinal epithelial damage exposes immune cells to gut microbes and triggers an inflammatory response, which is the basis for cancer development. In the chronic inflammatory environment, cells produce excessive pro-inflammatory cytokines, including TNF-α, IL-17, IL-6, and IL-1β, which have critical roles in the development of CAC [25,26]. Similar to this study, earlier investigations have demonstrated that *L. gasseri* can reduce intestinal inflammation and the risk of colon cancer related to colitis by upregulating the expression of anti-inflammatory cytokines and downregulating the expression of pro-inflammatory cytokines [27]. According to our study findings, *B. bifidum* H3-R2 and *L. lactis* KLDS4.0325 may lessen the inflammatory response brought on by AOM/DSS and prevent the development of inflammation into CRC in mice by controlling the level of inflammatory cytokines. In addition, *B. bifidum* H3-R2 and *L. lactis* KLDS4.0325 reduced AOM/DSS-induced colon length shortening and weight loss in mice, which was related to their inhibitory effect on intestinal inflammation.

The NOD-like receptor protein 3 (NLRP3) inflammasome is essential for controlling intestinal homeostasis and generating an innate immune response. It is responsible for the proteolytic maturation and release of the pro-inflammatory cytokine IL-1β [28], which encourages the development of diseases connected to inflammation, including IBD and CAC [29]. In this work, the expressions of NLRP3, IL-1β, and caspase-1 were increased in mice treated with AOM/DSS. After cell stress, the inflammasome responsible for the maturation of IL-1β was activated and caused the activation of caspase-1 [30]. The combination of *B. bifidum* H3-R2 and *L. lactis* KLDS4.0325 reduced the expression of NLRP3, IL-1β, and caspase-1 genes, indicating that the combined strains could prevent colonic inflammation by inhibiting NLRP3 inflammasome-related signaling pathway genes.

HIF-1α promotes the polarization of regulatory T cells and aids tumor cell proliferation, hence facilitating the development and progression of CAC [31]. Therefore, inhibition of HIF-1α is one of the targets of antitumor therapy. The combination of *B. bifidum* H3-R2 and *L. lactis* KLDS4.0325 could reduce the increase in HIF-1α content caused by inflammation. Zhou et al. discovered that *Saccharomyces boulardii* may prevent the progression of the epithelial–mesenchymal transition by treating mice with DSS-induced colitis to reduce the expression of HIF-1α [32]. In addition, patients with CRC had higher levels of HIF-1α in their peripheral blood, the tumor microenvironment, and tumor-draining lymph nodes [33].

Proteins associated with tight junctions influence intestinal permeability and the development of CAC [34,35]. ZO-1 and claudins maintain the integrity of the intestinal barrier and regulate its permeability. Occludin may also play a role as a signaling protein in inflammation, cell differentiation, and proliferation [36]. In this work, the combination of *B. bifidum* H3-R2 and *L. lactis* KLDS4.0325 reduced ZO-1, occludin, and claudin damage in CAC mice. MPO levels decreased due to the restoration of the intestinal epithelium and the reduction of pro-inflammatory cytokines. *B. bifidum* H3-R2 and *L. lactis* KLDS4.0325 were found to have a protective effect against CAC. The current findings are in accordance with those of previous research showing that MPO activity is decreased in UC patients who take *L. delbrueckii* and *L. fermentum* preparations [37].

Chronic intestinal inflammation and colorectal cancer are both linked to an imbalance in the intestinal microbiome [38]. The combination of *B. bifidum* H3-R2 and *L. lactis* KLDS4.0325 increased the abundance of Lactobacillaceae. *Lactobacillus* species are thought to be useful for digestive diseases such as IBD [39], and studies have indicated that *Lactobacillus* can prevent colitis and colorectal cancer in mice [40,41]. *Akkermansia* is a Gram-negative anaerobe that has been identified as a promising probiotic candidate [42]. *Akkermansia* is less prevalent in individuals with metabolic impairment, according to clinical studies [43]. However, some studies have demonstrated that the relative prevalence of *Akkermansia* is greater in CRC patients than in healthy individuals [44,45]. By raising early inflammation levels and intestinal epithelial cell proliferation in mice, Wang et al. discovered that *Akkermansia muciniphila* may promote the growth of CRC [46]. Similar to earlier findings, this experiment showed an increase in the relative abundance of Akkermansiaceae in the model group. According to studies, a diet high in carbohydrates increases Prevotellaceae in the gut, which is linked to the production of secondary compounds, like hydrogen sulfide, which can damage DNA and produce reactive oxygen species, as well as the development of colorectal cancer [47]. High levels of Prevotellaceae were also detected in stool and tumor samples of CRC patients [48]. This study demonstrated that Akkermansiaceae and Prevotellaceae abundances in the colon contents of CAC mice could be significantly reduced by administering a combination of *B. bifidum* H3-R2 and *L. lactis* KLDS4.0325 and that this combination could also improve intestinal microbial disorders to have a significant impact on CAC prevention.

## 5. Conclusions

The newly developed combination of *B. bifidum* H3-R2 and *L. lactis* KLDS4.0325 may inhibit the proliferation of colon cancer cells and promote cell apoptosis as well as alleviate CAC symptoms by regulating intestinal flora, improving intestinal barrier integrity, and lowering inflammation. The combination of the two strains had a greater effect than each strain alone. Therefore, *B. bifidum* H3-R2 in combination with *L. lactis* KLDS4.0325 has the potential to prevent and alleviate CAC.

## Data Availability

The original contributions presented in the study are included in the article, further inquiries can be directed to the corresponding author/s.
